# Pickleball and mental health in adults: A systematic review

**DOI:** 10.3389/fpsyg.2023.1137047

**Published:** 2023-02-21

**Authors:** Juan-Leandro Cerezuela, Maria-Jesus Lirola, Adolfo J. Cangas

**Affiliations:** ^1^Faculty of Education Sciences, University of Almería, Almería, Spain; ^2^Department of Psychology, University of Almería, Almería, Spain

**Keywords:** inclusive sport, physical activity, mental disorder, mental health, wellbeing

## Abstract

**Introduction:**

Physical activity has been extensively studied and numerous mental health benefits have been found. Pickleball is an emerging racquet sport, which is characterized by its accessibility to all audiences and has become especially popular in the United States among the elderly. It is a novel team game and its inclusive nature is innovative for health improvement. The purpose of this systematic review was to review and evaluate existing studies that have examined the effects of pickleball on the mental and psychological health of individuals.

**Methods:**

A systematic review was conducted on articles found in Scopus, PubMed, Elsevier, Web of Science (WoS), PsyINFO, Dialnet, and Elton B. Stephens Company (EBESCO) from 1975 to the present. The keywords used was a five combination between “Pickleball” joint with different terms by the connector AND, the second part of the combo could be “mental disorder” OR “anxiety” OR “depression” OR “psychological health” OR “mental health.” Eligibility criteria included: papers focused on pickleball, in English or Spanish, on mental health variables, without establishing an age range. We excluded duplicate works, without access or that did not address the objective of this study.

**Results:**

The search resulted in 63 papers, of which 13 were selected. A total of 90.74% of the population were people over 50 years of age. The results show significant improvements in the different psychological variables measured in pickleball practitioners: personal wellbeing, life satisfaction, depression, stress, happiness, etc., pickleball shows potential as a new tool to work and improve people’s mental health.

**Conclusions:**

The pickleball is displayed as an inclusive sport that does not need adaptations, resulting of great interest to be worked in different populations with mental problems.

## Introduction

Physical activity and sport have been widely studied, given the benefits in physical health and fitness improvement that arise from their practice (Granero-Gallegos et al., 2020; Medellín Ruiz et al., 2020; Manresa-Rocamora et al., 2021). However, physical-sport activity has also been shown to be a useful tool for achieving benefits in people’s mental health (White et al., 2017; Kandola et al., 2019). Chillón-Garzón et al. (2002), understand physical-sport activity as a physical activity whose objective is oriented toward health, moving away from sporting performance. Given the wide-ranging benefits it offers, it is of great interest to look for new practices that are innovative in order to encourage the greatest possible number of people to take part in physical sport. Pickleball is an emerging sport that has experienced exponential growth in the United States in recent years. Although so far it has been most popular among veteran athletes and elderly people (Ryu et al., 2018; Vitale and Liu, 2020), it has characteristics that make it possible for all ages to practice, suggesting the potential for working on the benefits that this emerging sport modality can have on mental health.

### The psychological benefits of physical and sporting activity

The inclusive nature that pickleball intrinsically produces, leads us to consider the potential of this sport in the field of mental health, as recognized by the World Health Organisation Regional Office for Europe (2016). Physical activity and sport can play a fundamental role in improving social inclusion and adapting healthy lifestyles in people with mental health problems.

The population with mental disorders benefits from the practice of physical activity in order to break with stereotypes, design and implement mechanisms of action that break with the stigma that weighs on these people, which has a negative effect on their social relationships (Casado et al., 2020). It is necessary to propose different anti-stigma programmes that help to favor this community of people (Cangas et al., 2017; Gallego et al., 2020). In this sense, the practice of physical and sporting activity has a positive effect on the mental health of users (Silverman and Deuster, 2014). People with stress and depression benefit from the functioning of the brain and hormonal systems that are triggered during their practice, which is essential for the improvement of various mental disorders (Portugal et al., 2013). This population has its symptoms reduced through sporting activities that generate adherence to regular exercise (Deslandes et al., 2009). Furthermore, physical activity complements the different pharmacological treatments, alleviating symptoms (Blumenthal et al., 2007). However, as suggested by Lawrence et al. (2010) and Stubbs et al. (2016), people with various mental disorders such as schizophrenia may have a high prevalence of risk factors or pathologies: obesity, dyslipidaemia, smoking, hypertension, hyperglycemia, and physical inactivity that worsen their mental, physical, and social health. At the same time, they have a lower cardiorespiratory capacity than the rest of the population.

In the same vein, Mandini et al. (2022) reported that cognitive functions of people with schizophrenia were significantly related to guided walking programmes. In fact, walking has been indicated as an enjoyable activity with ameliorative outcomes for people with mental disorders, which highlights the resilience capacity to resist and cope with problems and demands by demonstrating an attitude of coping (Schetter and Dolbier, 2011). This resilience is closely linked to physical activity and psychological distress, stress, or anxiety, which can inform the design of interventions to improve mental health through physical activity (To et al., 2022). Training and socio-educational sports programmes can favor the recovery and social inclusion of people with mental health problems, generating an environment of healthy relationships at different levels (Cangas et al., 2019).

Although the psychological benefits of physical-sporting activity have been notably confirmed, however, its benefits using new sporting practices have not yet been analyzed. One of the physical-sporting practices that has been booming in recent decades has been pickleball. This practice is characterized by its great versatility and applicability to all types of participants, which makes it extremely attractive for intervention programmes aimed at improving the psychological health of the individual.

### Origin and characteristics of pickleball

According to information obtained from the (United States Amateur Pickleball Association, 2022, p. 21), pickleball is a game created by Joel Pritchard (Senator of the State of Washington) together with Bell and McCallum in 1965 on Bainbridge Island. The idea for the sport was born out of a family need to design a game that would be fun and inclusive for all members of the family. After playing golf one summer Saturday, Joel Pritchard, and Bill Bell, a successful businessman, returned to the Pritchard home on Bainbridge Island, WA (near Seattle) to find their families sitting around with nothing to do. The property had an old badminton court, so Pritchard and Bell searched for badminton equipment and couldn’t find a complete set of rackets. They improvised and started playing with ping-pong paddles and a perforated plastic ball. At first they set the net at a badminton height of 60 inches and volleyed the ball over the net. As the weekend progressed, the players found that the ball bounced well off the asphalt surface and soon lowered the net to 36 inches. The following weekend, Barney McCallum was introduced to the game at Pritchard’s house. Soon, the three men were creating rules, based largely on badminton. They did not lose sight of the original goal, which was to provide a game that the whole family could play.

Joan Pritchard invented the name “pickle ball,” a reference to the nautical term “pickle boat,” which refers to the last boat to finish a race. However, it has been popularly believed that the name comes from Pritchard’s family dog, a Cocker Spaniel named Pickles, whose fondness for the ball inspired the owner to name the sport after it. While it is true that this family had a dog named Pickles, the sport was created and named a few years before the animal became part of the family, and the sport was named after him.

**FIGURE 1:**
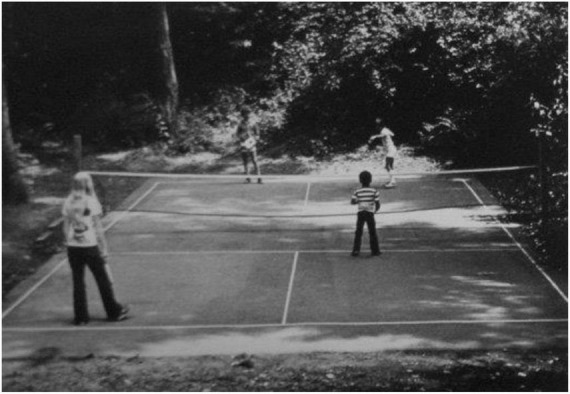
Original pickleball track. USAPA (United States Amateur Pickleball Association, 2022).

The first permanent pickleball court was built in the backyard of Bob O’Brian, Joel Pritchard’s friend and neighbor (Figure 1).

In the spring of 1976 the world’s first known pickleball tournament was held at the South Center Athletic Club in Tukwila, Washington. David Lester won the men’s singles and Steve Paranto came second. Many of the participants were college tennis players who knew very little about pickleball.

The United States Amateur Pickleball Association was organized to perpetuate the growth and advancement of pickleball nationally. The first rules were published in March 1984.

**FIGURE 2:**
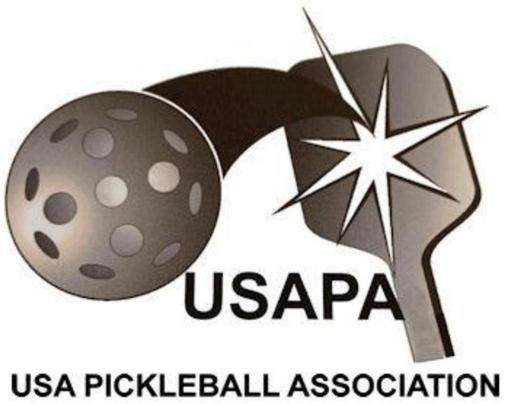
Logo of the United States Amateur Pickleball Association. USAPA (United States Amateur Pickleball Association, 2022).

The game of pickleball was first introduced at the Arizona Senior Olympics (United States Amateur Pickleball Association, 2022) thanks to the efforts of Earl Hill. The tournament was played at Happy Trails RV Resort in Surprise (United States Amateur Pickleball Association, 2022; Figure 2).

**FIGURE 3:**
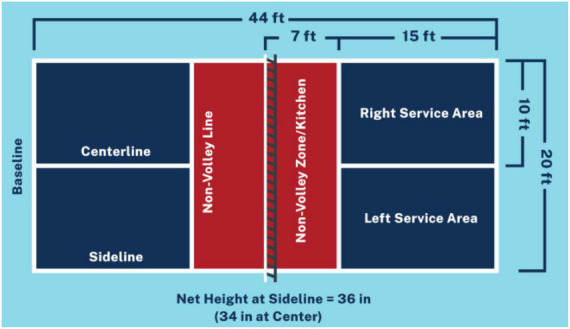
Dimensions of the pickleball court. USAPA (United States Amateur Pickleball Association, 2022).

The game is a combination of tennis, table tennis, and badminton. It is mainly played as a doubles game, but can also be played as a singles game. A small plastic ball with holes in it is used. This ball reaches a lower speed than other racket sports during the game. Players hit the ball with a racket or paddle whose standard size is 40 cm × 20 cm. They are larger than those used in table tennis, but smaller than those used in paddle tennis. It is played on a court similar to a badminton court. Its measurements are 6.1 m wide × 13.41 m long. With a net 86.36 cm high, arranged in the centre of the court in the same way as tennis. One of the main characteristics of this game is the presence of an area of the court, known as the non-volley or kitchen area (see Figure 3). Players may not hit the ball directly into this area unless they have previously voted. Matches are played to 11 points, and must be won by two points difference. To score, the player must win the point during his service [International Federation of Pickleball (IFP), 2022].

Pickleball does not place as much emphasis on the technical gesture as other racquet sports, but maintains a greater strategic and tactical element (Michael and Webster, 2020), which translates into a skills-based learning model (Bunker and Thorpe, 1982). One of the fundamental skills in this sport is the Dink, which consists of placing the ball in the opponent’s kitchen, with the aim of making it difficult for the opponent to attack (Zagrodnik, 2019). Due to the speed of the ball, it allows working on speed, agility, strategy, control and coordination in a very balanced way, positively involving cardiopulmonary capacity along with muscular condition (Wasem, 1994). Given these characteristics, pickleball is an easy sport to practice and accessible to all audiences. It is both economical and quick to set up on any hard court (Wray et al., 2021).

Research has repeatedly noted the psychological benefits of physical exercise (Hosker et al., 2019; Rodriguez-Ayllon et al., 2019). However, the field of physical exercise is a reality in flux and new practices are constantly emerging that aim to bring its use closer to the individual. One of the new practices is pickleball, which has the potential to work on and improve the mental health of its practitioners. Therefore, the aim of this paper is to review and evaluate existing studies that have examined the relationship between pickleball and the mental and psychological health of individuals.

## Materials and methods

### Location of studies

For the elaboration of this systematic review, it has been followed the principles set out in PRISMA statement (Moher, 2009), updated by Page et al. (2021). A literature review was conducted in the following databases: Scopus, PubMed, Elsevier, Web of Science (WoS), PsyINFO, Dialnet, and Elton B. Stephens Company (EBESCO) (including CINAHL, PsicoDoc, ERIC, Education Source and Medline), in order to compile different research studies written about pickleball between January 1975 (the year in which this sport began to expand) and December 2022.

The keywords used was a five combination between “Pickleball” joint with different terms by the connector AND, the second part of the combo could be “mental disorder” OR “anxiety” OR “depression” OR “psychological health” OR “mental health,” limiting the search to texts written in Spanish and English. The review of the information was completed with the bibliographic references found in the previous searches.

### Eligibility criteria

Considering the inclusion and exclusion criteria defined below for this study, the articles/studies were reviewed in two phases. Firstly, the title and abstract of the papers were reviewed and, secondly, the full text was reviewed.

#### Inclusion criteria

The publications located in the literature search were considered eligible if they met the following criteria: (a) papers focusing exclusively on pickleball; (b) only papers published in English or Spanish, as these are the languages known to the author of this paper; (c) papers that, at least in some of their variables, address mental, and psychological health; (d) empirical articles, including both observational and experimental studies; (e) as well as qualitative studies; (f) no age range of the participants was established for the selection of the articles; (g) the papers were not considered eligible for inclusion in the literature search.

#### Exclusion criteria

The located works were excluded based on some of the following criteria: (a) duplicate works; (b) works that had only an abstract; (c) works whose access was not possible through the databases or the request to the author/s; (d) works whose content was didactic programming on this sport or any other content that did not include an empirical study; (e) works that did not address the relationship established in the objective of this study; (f); works that included pickleball in multi-sport programmes; (g) works that did not include pickleball in multi-sport programmes.

### Data coding procedure

Information was extracted from each study and coded with the following structure:

• Author (name of first author and year of study).
• Number of participants and gender.
• Age of participants.
• Type of study.
• Instruments.
• Variables.
• Results found.

In case of observational and longitudinal studies based on an intervention, the weeks of intervention, description of the programme, duration, and frequency were extracted.

### Quality index of selected studies

In order to guarantee a higher methodological quality of the reviewed and selected studies, a quality index based on four items belonging to the Strengthening the Reporting of Observational Studies in Epidemiology (STROBE) Statement (Von Elm et al., 2007) was developed and applied. Firstly, the objective stated in the papers is analyzed by analyzing whether they indicate specific objectives, as well as the inclusion of any pre-established hypotheses. Secondly, the involvement of the participants is established, taking into account whether the selected papers clearly state the reason for sample selection, age and gender. A higher score is given for longitudinal studies than for cross-sectional studies in order to positively assess the involvement and impact of studies that follow up over time. Finally, we took into account whether the studies analyzed variables in a clear and well-established way, clearly defining outcomes, exposures and predictors, controlling for possible confounding, and effect-modifying variables (see Table 1).

**TABLE 1:** Quality index of included studies.

	Item	Criteria	Rating
1	Objective	Indicates specific objectives and hypotheses	1
2	Participants	Defined	1
		Not defined	0
3	Study type	Experimental	3
		Quasi-experimental	3
		Longitudinal	2
		Cross-sectional	1
		Correlational	1
		Qualitative	1
		Descriptive	0
4	Variables	Clearly described	1
		Not described	0

## Results

**FIGURE 4:**
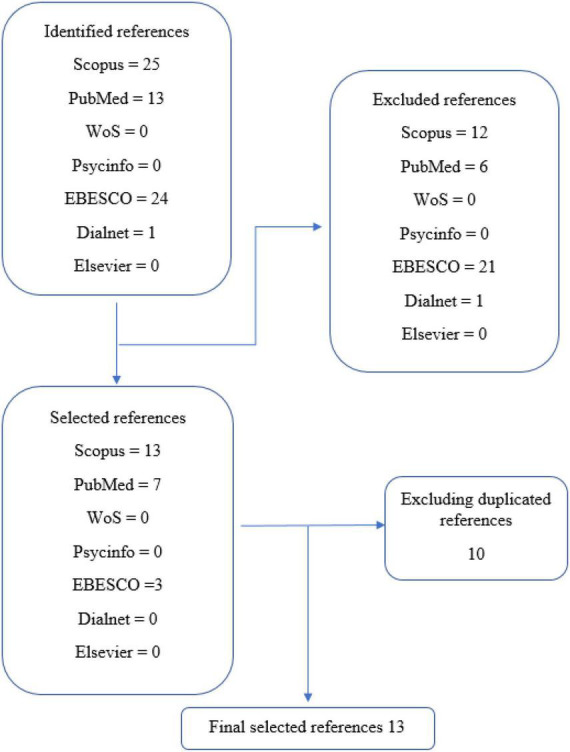
Flow chart.

Given the limited literature on this sport, only 63 articles were found from all the databases reviewed. After discarding all papers that did not meet the inclusion/exclusion criteria, the total number of studies was reduced to 13. The type of studies found were mostly quantitative, observational, cross-sectional, and correlational. Only three longitudinal studies were found, of which only one was quasi-experimental. No qualitative articles were found. Figure 4 shows the flowchart of the literature search carried out.

### Quality assessment

The quality assessment of the included studies (Figure 4) was performed on a quality scale based on four items belonging to STROBE (Von Elm et al., 2007). The overall mean score of the quality index of the included studies was 4.15 out of a possible total score of 6.

Table 1 shows the established criteria with the assigned score.

Table 2 shows the score obtained by each of the studies.

**TABLE 2:** Quality of studies.

References	Quality
Reynolds et al., 2016	5
Heo et al., 2018a	4
Ryu et al., 2018	4
Heo et al., 2018b	4
Gill and Swartz, 2019	2
Ryu et al., 2018, 2020	4
Kim et al., 2020	4
Casper et al., 2021	5
Kim et al., 2021	4
Wray et al., 2021	6
Buzzelli and Draper, 2020	4
Casper and Jeon, 2019	4
Chen et al., 2021	4

### Overview of the reviewed studies

Table 3 provides an overview of the different interventions reviewed with information on different variables of interest (e.g., number of women and men, age, type of study, instruments, variables, and outcomes) for each of the 13 articles reviewed. Of the 13 papers reviewed, we found eight correlational studies measuring the association between two or more psychological variables. One observational study based on a tournament conducted over time with a final collection of data for analysis is analyzed. A cross-sectional study examining the effects of pickleball on task orientation and ego. The total study population is 5,405 participants, with 4,905 people >50 years old (90.74%). Of which 761 men and 723 women have been identified.

**TABLE 3:** Characteristics of the reviewed studies.

References	Participants (*N*; gender)	Sample characteristics; age	Type of study	Variables	Instruments	Results found
Reynolds et al., 2016	*N* = 48; 24 older adults; 24 students	>65 years; undergraduates	Intergenerational pickleball tournament for a duration of 3 months	Intergenerational inclusion	Two qualitative feedback from students	Positive impact of pickleball tournament on intergenerational inclusion.
Heo et al., 2018a	*N* = 153; M: 63; W: 90	Pickleball players; >50 years old	Correlational study	Personal wellbeing loneliness Serious leisure Life satisfaction	SWB: SWLS SLIM Loneliness scale	Pickleball as leisure predicts SWB and quality of life, *p* < 0.01.
Ryu et al., 2018	*N* = 153; M: 63; W: 90	Pickleball players; >50 years old	Correlational study	Life satisfaction Social integration Optimism	SWLS LOT Social integration	Higher SWLS in retirees vs. non-retirees who play pickleball. Higher social integration in women, *p* < 0.05.
Heo et al., 2018b	*N* = 153; M: 63; W: 90	Pickleball players; >50 years old	Correlational study	Depression Serious Leisure Social Integration Optimism	MDI SLIM Social integration LOT	Negative correlation between depression and serious leisure in pickleball players, *p* < 0.01.
Gill and Swartz, 2019	Tuesday league *N* = 135; Monday league *N* = 166; M: 84; W: 82	Active pickleball players belonging to Tuesday’s men league and Monday mixed league	Proposed sport model Descriptive study	Strong bond	Model formula on weak and strong ties	Pickleball is a sport with strong team bonds.
Ryu et al., 2020	*N* = 208; M: 112; W: 96	Pickleball players; 50–83 years old	Correlational study	Authenticity Stress Happiness	Authenticity inventory PSS-4 Happiness	Authenticity significantly associated with reduced perceived stress and greater happiness, *p* < 0.01.
Kim et al., 2020	*N* = 208; M: 112; W: 96	Pickleball players; 50–83 years old	Correlational study	Level of participation pickleball Social capital Happiness	SLIM Perceived social capital questionnaire Happiness	Pickleball participation and perceived social capital predict perceived happiness, *p* < 0.001.
Casper et al., 2021	*N* = 36	Pickleball players; >65 years old	Observational and longitudinal study pre-COVID and during COVID	Level of participation pickleball Social connections Personal wellbeing	Sports participation Social connections SF-12 ULS-4 SWLS	Lower participation in pickleball correlates with lower life satisfaction, *p* < 0.05.
Kim et al., 2021	*N* = 208; M: 112; W: 96	Pickleball players; 50–83 years old	Correlational study	Level of participation pickleball Social capital Happiness	Sports participation Social connections Happiness	Participation and social connections predict happiness. *p* < 0.001. Connections predict participation, *p* < 0.001.
Wray et al., 2021	*N* = 20; M: 17; W: 3	Rural adults; 50–75 years	Longitudinal study Quasi-experimental 6 weeks three classes/week of 60 min. Pre-test and post-test.	Muscle function Cognitive function Pain Cardio metabolic risk	Hand-held dynamometer High jumps Neuropsychological tests NPRS HRV Interviews	Improvements in vertical jump height, *p* < 0.01. Cognitive performance and self-reported pain, *p* < 0.05.
Buzzelli and Draper, 2020	*N* = 3,012	Mage = 63.17 years; 3 years of pickleball experience	Estudio transversal	Sport motivation Task orientation Ego orientation	SMS Task and ego orientation in sport questionnaire QIRS	Participants reported being more task-oriented than ego-oriented in pickleball.
Casper and Jeon, 2019	*N* = 690	>55 years old	Cross-sectional study	Conscientiousness, attraction, attachment, and loyalty	PCM Sport questionnaire	Longer pickleball playing time is associated with higher PCM, *p* < 0.01.
Chen et al., 2021	*N* = 215; M: 135; W: 80	Pickleball players; <20 years old: 39; 21–30 years old: 73; 31–40 years old: 34; 41–50 years old: 29; 51–60 years old: 25; >61 years old: 15	Correlational study	Leisure participation Leisure satisfaction Personal wellbeing	Satisfaction, leisure participation, and wellbeing questionnaire Wellbeing	Satisfaction has significant relationship with wellbeing and leisure participation. *p* < 0.001 in pickleball players. Leisure participation is not significantly related to wellbeing in pickleball players.

SWLS, satisfaction with life scale; SLIM, serious leisure inventory measures; LOT, life orientation test; MDI, major depression inventory; PSS-4, perceived stress scale; SF-12, health survey; ULS-4, UCLA loneliness scale-4; NPRS, pain rating scale; SMS, sport motivation scale; TEOSQ, task and ego orientation in sport questionnaire; QIRS, quality and importance of recreational services; PCM, psychological continuum model (Competition, Fun, Fitness, Skills Mastery, and Socialization); HRV, heart rate variability.

Of the total papers analyzed, three of them conducted a longitudinal study (Reynolds et al., 2016; Casper et al., 2021; Wray et al., 2021) where the evolution of the different variables established at different points in time was observed. Of these three, the most striking study, despite presenting a smaller study sample, is the pickleball-based intervention by Wray et al. (2021). This study applied a 6-week programme in a rural population with a high level of sedentary lifestyles, in order to measure different physical and psychological fitness variables. They showed highly significant improvements in jumping ability, cognitive performance, and self-perceived pain.

On the other hand, different studies (Heo et al., 2018a,b; Ryu et al., 2018) correlated, through self-reported questionnaire responses, life satisfaction, serious leisure, life orientation, loneliness, social integration, and major depression in elderly people who were accustomed to playing pickleball. They found that this sport focused on serious leisure was negatively correlated with depression, increased life satisfaction, decreased loneliness, increased quality of life, and suggested greater social integration, which in turn was greater in women. Serious leisure is understood as “the systematic pursuit of a basic amateur, hobby or volunteer activity that people find so substantial, interesting and satisfying that, in the typical case, they embark on a (leisure) career focused on acquiring and expressing a combination of their special skills, knowledge and experience” (Stebbins, 2016, p. 5).

Furthermore, Kim et al. (2020), Ryu et al. (2020), and Kim et al. (2021), analyzed the relationships between perceived stress, serious leisure, authenticity, participation and social capital with happiness in older pickleball players. The results of the study suggest that participation, social capital, and leisure predict happiness. At the same time, happiness and authenticity decrease perceived stress.

Regarding the studies that were conducted by Buzzelli and Draper (2020), together with that of Casper and Jeon (2019), they stand out for presenting the highest samples, specifically with samples of 3,012 and 690 participants, respectively. Buzzelli and Draper (2020) highlighted the innovative approach of pickleball compared to other competitive sports. They emphasized task mastery and skill mastery over ego. In general, participants in this study reported being more intrinsically motivated than extrinsically motivated and, in particular, were more motivated by a sense of accomplishment and reported being more task-oriented. Casper and Jeon (2019) in turn, highlighted the advantages of increasing practice time to enhance competition, enjoyment, fitness, skill mastery, and socialization.

Finally, we find, on the one hand, the study conducted by Gill and Swartz (2019) on a proposed sports model. This model seeks to analyze the possible benefits of pickleball as a team sport where its members form a strong bond. They establish a model formula on weak and strong bonds to perform this analysis. On the other hand, the study by Chen et al. (2021), which introduces the variable of wellbeing. In this study they found a positive correlation with satisfaction toward leisure.

## Discussion

The aim of this research was to review the studies that analyze the relationship between pickleball and the mental health of the people who practice it. To this end, 13 studies were selected that met the established inclusion criteria. The participants in these studies were 90.75% older people, aged between 50 and 83 years. Practically all of the studies found were carried out on this sector of the population. This suggests the possibilities of generating reinsertion and physical-sports habits which, as analyzed by Lera-López and Suárez-Fernández (2019) tend to be abandoned as age increases. In this sense, pickleball, given its ease of play and low-impact nature, can be an enjoyable way for people of all ages to stay active and fit and help promote a healthy lifestyle (Changstrom et al., 2021). This results in more effective and enjoyable physical activity for populations with more barriers to accessing physical sporting activity, thus promoting healthy sporting activity, which does not generate stress and trigger frustrations and a range of mental disorders resulting from high competition (Armstrong and VanHeest, 2002; Schaal et al., 2011).

Various sports programmes such as the one analyzed by Walter et al. (2021), despite showing short-term benefits in different psychological disorders such as depression or anxiety, soon after the end of the programme they worsen again. On the contrary, pickleball is shown to be a sport with great inclusive and intergenerational possibilities (Reynolds et al., 2016), which is of great interest for its application in people with mental disorders. The studies analyzed reflect the great importance and potential impact of pickleball in this field. On the one hand, personal wellbeing and life satisfaction, showing itself as a promoter of health effects and on the other hand, Pickleball as an agent with preventive effects by reducing depression symptoms, indicating high values in its participants in both directions. Similarly, to other physical-sport activities, where higher frequency of physical activity leads to increased mental health (Murphy et al., 2022; Nweke et al., 2022; Shaw et al., 2022).

In addition, as suggested by numerous authors such as Greiner (2019), Quail (2019), Forrester (2020), Vitale and Liu (2020), Weiss et al. (2021), Atkinson et al. (2022); pickleball is a sport-physical activity with a very low prevalence of accidents and injuries. This means that it is a safe sport with few risks to people’s health.

The interest in bringing pickleball to education has not been non-existent, but it has been very discreet, with different proposals spread out over time. Works such as those of Davis (1976), Wasem (1994) or more recently Michael and Webster (2020). This is striking, given that this sporting practice is of interest in the field of education as a new tool for working with students. Physical education should be shown as a subject in favor of social inclusion, of a socializing nature without excluding anyone on the basis of age, gender, functional diversity, culture, ethnicity or race. It should be a tool for inclusion that reaches the most excluded sectors (Levoratti and Zambaglione, 2015). Given the need to implement measures that encourage new forms of personal constructions that respect diversity, improving personal potential (Águila et al., 2020), new skills and new knowledge are acquired that help to know oneself, where each member interacts and contributes their personality regardless of the capabilities of each person. Everyone must participate equally. Work through interventions that improve diversity and inclusion (Lirola, 2020; Lirola et al., 2020).

Similarly, the results seem to suggest the inclusive possibilities regarding gender in this sport. Unlike what happens in most physical-sports activities, where women have less presence and participation and the gender role is more pronounced (Vicente-Pedraz and Paz Brozas-Polo, 2017; Piedra, 2019). The data seem to indicate a greater participation equality between men and women in the habitual practice of pickleball. Of the total sample of studies, 761 men and 723 women were identified as playing pickleball. This may be largely due to the very characteristics of this game that make it so peculiar and suitable for all audiences, together with a regulation that encourages mixed play in a completely natural way, unlike other racket sports where women encounter numerous barriers during its practice (Ferrer Suárez et al., 2022).

As limitations of this study, having used specific search tools (Scopus, PubMed, WoS, Psychoinfo, Elsevier, Dialnet, and EBSCO) there is a possibility of loss of research articles. Mention the need to analyze studies in languages other than those known to the author, if any. It should also be added that the sample analyzed in the studies is completely limited to older people, so it would be necessary to consider new studies in populations with different characteristics. Finally, it is necessary to mention that most of the articles reviewed were correlational studies. There is a notable lack of longitudinal, experimental, and qualitative studies examining the effects of this physical-sports practice. This is certainly a clear difference with existing studies in other racquet sports, where several longitudinal studies can be found (Gutiérrez-Álvarez et al., 2022; Li, 2022; Martínez-Gallego et al., 2022). Therefore, the existing studies to date examining the relationship between pickleball and mental health suggest certain associations between both variables, but no clear benefits of pickleball practice on the mental health of people who practice this sport can be attributed. This indicates that we are dealing with a field that is emerging at the moment, a new field that poses the need to carry out new lines of research of an experimental and qualitative nature, in order to cover the possibilities of this new modality.

## Conclusion

The review carried out in this study shows the potential of pickleball as a new tool to work on and improve people’s mental health. This sport is very striking, because from its own conception and nature it becomes an inclusive sport that does not need adaptations to be attractive to anyone. The results of the studies reviewed show associations between the use of pickleball and psychological variables; however, cause-effect relationships between both constructs cannot be established given the scarcity of experimental and longitudinal studies. This review makes clear the need to continue exploring the possibilities of this new game, still largely unknown, but which is gradually making its way into our society. Given the possibilities and applications, future studies should clarify the role that pickleball can play in improving mental health. Thus, the results of future studies should determine to what extent this sport is of interest to be worked in populations with mental disorders, given the accessibility of this sport, which can be fun and innovative, helping to generate healthy and active habits in a population that tends to go more unnoticed by society. This review, with its possible limitations, should only be seen as a starting point for future research related to the field of physical activity, sport, and mental health.

## Data availability statement

The original contributions presented in this study are included in the article/supplementary material, further inquiries can be directed to the corresponding author.

## Author contributions

AC: conceptualization, visualization, and supervision. J-LC and M-JL: methodology. M-JL and AC: validation and resources. J-LC: investigation and writing—original draft preparation. M-JL: writing—review and editing. All authors read and agreed to the published version of the manuscript.
